# Precisamos Falar de Determinantes Sociais de Saúde Cardiovascular

**DOI:** 10.36660/abc.20230569

**Published:** 2023-10-16

**Authors:** Humberto Graner Moreira

**Affiliations:** 1 Universidade Federal de Goiás Goiânia GO Brasil Universidade Federal de Goiás – Liga de Hipertensão Arterial, Goiânia, GO – Brasil; 2 Hospital Israelita Albert Einstein Unidade de Pronto Atendimento Goiânia GO Brasil Hospital Israelita Albert Einstein – Unidade de Pronto Atendimento, Goiânia, GO – Brasil

**Keywords:** Doenças Cardiovasculares/complicações, Fatores de Risco, Serviços de Saúde Comunitária, Fatores Socioeconômicos, Estatística de Saúde, Mortalidade

Além dos fatores de risco biológicos tradicionais, há uma série de determinantes sociais que desempenham um papel significativo no risco cardiovascular. Os Determinantes Sociais de Saúde (DSS) referem-se às condições sociais, econômicas, culturais, étnicas, educacionais e ambientais em que as pessoas vivem e como essas condições impactam na saúde. Estes determinantes podem incluir fatores como renda, condições de trabalho, acesso aos serviços de saúde, educação, habitação, meios de transporte, redes de apoio, entre outros.^1,2^

A relação entre fatores socioeconômicos e saúde começa a ser observada por ocasião da Revolução Industrial na Europa. Um dos pioneiros nesse campo foi Rudolf Virchow, médico e político alemão, que, em meados do século XIX, destacou o impacto das condições sociais na saúde. Ele enfatizou que para melhorar a saúde da população, era necessário abordar questões como educação, habitação e trabalho. É de Virchow a frase “a medicina é uma ciência social, e a política nada mais é do que uma medicina em grande escala”.^3^

Mas, o conceito de DSS como o entendemos hoje, começou a tomar forma no século XX. A identificação de fatores de risco cardiovasculares trouxe também a informação de que determinados grupos populacionais estavam expostos de forma diferentes aos mesmos. Da mesma forma, as desigualdades sociais e a baixa escolaridade foram reconhecidas como condições adicionais associadas com maior mortalidade por doenças cardiovasculares (DCV).^2,4^

Estudos anteriores já haviam demonstrado que a mortalidade por DCV - incluindo doenças isquêmicas do coração (DIC) e cerebrovasculares (DCBV) – têm uma incidência desigual de acordo com a região do país.^5,6^ Uma análise recente da tendência temporal de mortalidade por DCV no Brasil revelou que a região Nordeste apresentou aumento nas taxas de óbitos, ao passo em que as regiões Sul e Sudeste tiveram reduções nas mesmas.^6^ A discussão sobre desigualdades sociais e econômicas regionais sempre fundamentou esses achados, mas os mesmos eram inferidos.

O estudo de Bichara et al.^7^ sobre a mortalidade por DIC e DCBV no Brasil, conduzido no período de 2000 a 2019, fornece uma visão abrangente e reveladora sobre a influência dos determinantes sociais na saúde cardiovascular no país. Além das conhecidas taxas de mortalidade por essas doenças no Brasil, os autores trazem para a discussão o índice de vulnerabilidade social (IVD) e o índice de desenvolvimento social (ISD), iluminando como eles interagiram com a incidência de óbitos por DCV em diferentes estados e regiões.

A constatação de que houve melhora desses índices sociais no período estudado endossa o crescimento econômico do país no período. Quando se analisaram as variações percentuais das taxas de mortalidade padronizadas por DIC e DCBV com os índices sociais, observou-se que os estados com melhores indicadores foram também aqueles que apresentaram maior redução proporcional da mortalidade. Um fato relevante é que, mesmo estados das regiões Norte e Nordeste que evidenciaram uma melhora significativa nos IDS e IVS, não necessariamente apresentaram redução da mortalidade por doenças cardiovasculares. Isso sugere a possibilidade de que talvez haja um limiar de desenvolvimento social mínimo que precisa ser obtido para que os efeitos na redução da mortalidade sejam percebidos.

Embora tenhamos avançado em relação à discussão das estatísticas cardiovasculares no cenário nacional, inclusive com dados atualizados anualmente, ainda ignoramos DSS.^8^ Em parte, porque ainda faltam pesquisas mais profundas de como estes determinantes interagem com os diversos outros fatores de risco. Mas, também, porque talvez atribuamos (erroneamente) a responsabilidade desses tópicos exclusivamente aos gestores de saúde.

No entanto, é cada vez mais evidente que o médico precisa identificar aqueles determinantes sociais desfavoráveis durante sua tradicional anamnese clássica. Isto inclui: rendas precárias, acessibilidade e qualidade da educação, disponibilidade de alimentos nutritivos, perspectivas de emprego, meios de transporte utilizados, ambiente de moradia. Mesmo que não possamos modificar a realidade de muitos daqueles pacientes, é possível oferecer soluções em saúde que possam mitigar seus efeitos, uma vez reconhecidos. Esta também é uma das principais mensagens do recente posicionamento da *American Heart Association* sobre os determinantes sociais e os desfechos cardiovasculares.^9^ Além de fornecer uma revisão abrangente do impacto das determinantes sociais na saúde cardiovascular, o documento também faz um apelo à comunidade médica e aos gestores para a necessidade de reconhecer e abordar os determinantes sociais como uma parte integrada da prevenção e tratamento das DCV.

O estudo de Bichara et al.^7^ acrescenta um novo nível de compreensão das nuances do impacto das determinantes sociais na saúde cardiovascular no Brasil. Seus achados sinalizam para um chamado à ação em políticas públicas mais inclusivas e abordagens clínicas que considerem as determinantes sociais como componentes centrais para mitigar o fardo das doenças cardiovasculares.
