# Supplementary material for: Precisamos Falar de Determinantes Sociais de Saúde Cardiovascular
Source: Arq Bras Cardiol. 2023 Oct 16;120(8):e20230569. [Article in Portuguese] doi: 10.36660/abc.20230569 (PMC10586820; doi:10.36660/abc.20230569)
Supplement: Translation

# Translation: We Need to Talk about Social Determinants of Cardiovascular Health

**Authors:** Humberto Graner Moreira

**Affiliations:** 1 Brazil Universidade Federal de Goiás – Liga de Hipertensão Arterial, Goiânia, GO – Brazil; 2 Brazil Hospital Israelita Albert Einstein – Unidade de Pronto Atendimento, Goiânia, GO – Brazil

**Keywords:** Cardiovascular Diseases/complications, Risk Factors, Community Health Services, Socioeconomics Factors, Health Statistics, Mortality

In addition to traditional biological risk factors, several social determinants play a significant role in cardiovascular risk. The Social Determinants of Health (SDH) refer to the social, economic, cultural, ethnic, educational, and environmental conditions in which people live and how these conditions impact health. These determinants can encompass factors such as income, working conditions, access to health services, education, housing, transportation, support networks, and others.^1,2^

The relationship between socioeconomic factors and health began to be observed during the Industrial Revolution in Europe. One of the pioneers in this field was Rudolf Virchow, a German physician, and politician who, in the mid-nineteenth century, highlighted the impact of social conditions on health. He emphasized that addressing issues such as education, housing, and work was necessary to improve the population’s health. It is from Virchow the phrase “medicine is a social science, and politics is nothing but medicine on a large scale”.^3^

However, the concept of SDH as we understand it today began to take shape in the 20th century. The identification of cardiovascular risk factors also revealed that specific population groups were differentially exposed to these factors. Similarly, social inequalities and low educational levels were recognized as additional conditions associated with increased mortality from cardiovascular diseases (CVD)

Previous studies had already shown that CVD mortality - including ischemic heart disease (IHD) and cerebrovascular disease CeVD - have an uneven incidence according to the region of the country.^5,6^ A recent analysis of the temporal trend in CVD mortality in Brazil revealed that the Northeast region showed an increase in death rates, while the South and Southeast regions had reductions.^6^ The discussion on regional social and economic inequalities has always supported these findings, but they were just inferred.

The study by Bichara et al.^7^ on IHD and cEvd mortality in Brazil, conducted from 2000 to 2019, provides a comprehensive and revealing view of the influence of social determinants on cardiovascular health in the country. In addition to the known mortality rates for these diseases in the country, the authors bring to the discussion the Social Vulnerability Index (SVI) and the Social Progress Index (SPI), illuminating how they interact with the incidence of deaths from CVD in different states and regions.

The finding that there was an improvement in these social indices in the period studied supports the country’s economic growth over the same timeframe. When the percentage changes in standardized mortality rates for IHD and CeVD were analyzed with social indices, it was observed that the states with the best indicators were also those that exhibited a greater proportional reduction in mortality. A noteworthy fact is that even states in the North and Northeast regions that showed a significant improvement in the SPI and SVI did not necessarily show a reduction in mortality from cardiovascular diseases. This suggests the possibility that there may be a minimum threshold of social development that needs to be achieved for the effects on mortality reduction to be perceptible.

Although we have advanced concerning the discussion of cardiovascular statistics in the national scenario, including annually updated data, we still ignore SDH.^8^ In part because deeper research on how these determinants interact with the various other risk factors is still lacking. Additionally also because we may erroneously attribute responsibility for these topics exclusively to healthcare managers.

Nevertheless, it is increasingly evident that the physicians need to identify those unfavorable social determinants during their traditional classical anamnesis. These include poor incomes, accessibility and quality of education, availability of nutritious food, employment prospects, means of transport used, and housing environment. Even if we cannot change the reality of many of those patients, it is possible to offer healthcare solutions that can mitigate their effects once recognized. This is also one of the main messages of the American Heart Association’s recent scientific statement on social determinants and cardiovascular outcomes.^9^ In addition to providing a comprehensive review of the impact of social determinants on cardiovascular health, the document also appeals to the medical community and policymakers for the need to recognize and address social determinants as an integrated part of CVD prevention and treatment.

The study by Bichara et al.^7^ adds a new level of understanding to the nuances of the impact of social determinants on cardiovascular health in Brazil. Their findings signal a call to action on more inclusive public policies and clinical approaches that consider social determinants as central components to mitigate the burden of cardiovascular disease.
